# Assessing the Risk of Urinary Tract Infection and Invasive Bacterial Infection in Febrile Infants Aged 7-90 Days With COVID-19

**DOI:** 10.7759/cureus.82405

**Published:** 2025-04-16

**Authors:** Tara L Greenhow, Tran H Nguyen, Madeline J Somers, David R Vinson, Dustin G Mark, Patrick J Van Winkle, Mary E Reed, Daniel D DiLena, Adina Rauchwerger, Dustin W Ballard

**Affiliations:** 1 Pediatric Infectious Diseases, Kaiser Permanente Northern California, San Francisco, USA; 2 Pediatric Hospital Medicine, Kaiser Permanente Northern California, Roseville, USA; 3 Epidemiology and Public Health, Kaiser Permanente Northern California, Pleasanton, USA; 4 Emergency Medicine, Kaiser Permanente Northern California, Roseville, USA; 5 Emergency Medicine, Kaiser Permanente Northern California, Oakland, USA; 6 Pediatric Hospital Medicine, Kaiser Permanente Southern California, Anaheim, USA; 7 Research, Kaiser Permanente Northern California, Pleasanton, USA; 8 Emergency Medicine, Kaiser Permanente Northern California, San Rafael, USA

**Keywords:** bacteremia, bacterial meningitis, covid-19, febrile infant, urinary tract infection

## Abstract

Introduction

Concomitant urinary tract infection (UTI) or invasive bacterial infection (IBI) in previously healthy, well-appearing febrile infants with COVID-19 is low. We sought to review the rates of UTI and IBI in all febrile infants with COVID-19 presenting to community emergency departments (EDs).

Methods

We retrospectively reviewed infants aged 7-90 days with COVID-19 from July 1, 2020, to August 31, 2022, who had an ED visit. Infants without fever or with COVID-19 more than seven days prior to the index ED visit were excluded. We collected data on blood, urine, and cerebrospinal fluid (CSF) culture results. UTI, bacteremia, and bacterial meningitis were defined by culture review as per prior standards.

Results

We included 622 infants, of whom 329 were febrile. Older infants and those presenting later in the pandemic had lower rates of complete evaluation. Of the 201 infants with urine collected, four (2%) had a UTI. Of the 184 infants with blood cultures obtained, 19 (10.3%) had contaminated blood cultures. One of the 159 infants (0.6%) with both blood and urine collected had an *Escherichia coli* bacteremic UTI. Only 12 infants had CSF obtained; however, no infants received treatment for bacterial meningitis. Factors associated with UTI were higher white blood cell (p=0.001) and absolute neutrophil counts (p=0.036), and abnormal urinalysis (UA) and urine microscopy (p<0.001).

Conclusions

Febrile infants with COVID-19 are at low risk for UTI and even lower risk for IBI. We recommend, at a minimum, that all febrile well-appearing infants aged 7-60 days with COVID-19 be evaluated for concomitant bacterial infections with a UA with microscopy.

## Introduction

Infants less than 90 days of age with COVID-19 may present to the emergency department (ED) with a variety of symptoms such as poor feeding, respiratory distress, hypoxia, and fever without a source [1-5]. Febrile young infants with viral respiratory tract infections are known to have lower rates of concomitant urinary tract infections (UTIs) and invasive bacterial infections (IBIs) (e.g., bacteremia and bacterial meningitis) compared with febrile infants without viral respiratory infections [6]. This observation appears to extend to previously well infants less than 90 days with SARS-CoV-2 [3-5,7-16], even suggesting lower rates of IBI than seen with other viral infections [17]. Although the American Academy of Pediatrics (AAP) released a Clinical Practice Guideline (CPG) [18] in 2021, citing the low risk of IBI in infants with concomitant viral infections, the CPG did not provide guidance on the evaluation of the febrile infant with bronchiolitis and other viral infections.

There is a growing body of literature that previously healthy, well-appearing febrile infants with COVID-19 are at substantially lower risk for UTI and IBI. We aimed to broaden the scope of previous studies by examining the incidence of UTI and IBI in all febrile infants with COVID-19 who presented to any of 21 community EDs within a large Northern California integrated health care system.

This work was presented in part as an oral presentation at the Society for Academic Emergency Medicine Annual Conference on October 10, 2023.

## Materials and methods

The Kaiser Permanente Northern California (KPNC) Institutional Review Board approved this data-only observational study with a waiver of informed consent.

Study design

We retrospectively analyzed KPNC’s electronic health record (EHR) to identify all infants aged 7-90 days with COVID-19 (confirmed by diagnosis code or SARS-CoV-2 positive real-time reverse transcription-polymerase chain reaction (RT-PCR)) from July 01, 2020, to August 31, 2022, who received a COVID-19 diagnosis within seven days prior to or three days after an ED visit. Infants without fever (i.e., maximum temperature < 38°C inside or outside a medical facility) or with COVID-19 more than seven days prior to the index ED visit were excluded.

During the study period, SARS-CoV-2 testing was recommended in all febrile infants presenting to the ED and was required for all hospital admissions. Multiple platforms were used for real-time RT-PCR SARS-CoV-2 testing (Table 1). Home COVID-19 testing was not FDA approved for children less than two years of age; however, if an infant had epidemiologic risk for COVID-19 and reported a positive home COVID-19 test, this was considered evidence of COVID-19 infection (type of home test not available).

**Table 1:** Definitions of clinical findings, history, and laboratory studies RT-PCR: reverse transcription-polymerase chain reaction; WBC: white blood cell; hpf: high-powered field; UA: urinalysis; CFU/mL: colony-forming units per milliliter; CSF: cerebrospinal fluid

Clinical Findings	
Regular expression-based text parsing of clinical notes	Physician notes in the ED reviewed: history of present illness for maximum temperature measurement outside of a medical setting, and general or constitutional appearance to assess for ill appearance.
Fever	Any measured temperature ≥ 38°C inside or outside a medical setting.
Ill appearance	One of the following documented in a physician examination or assessment: “cold,” “decreased mental status,” “difficult to arouse,” “floppy,” “hypotonic,” “ill appearing,” “inconsolable,” “irritable,” “listless,” “toxic,” “nonresponsive,” “lethargic,” “poorly perfused,” “sick,” “shock.”
History	
Not previously well	Premature infants < 37 weeks gestational age; Infants with underlying neuromuscular, cardiovascular, respiratory, gastrointestinal, metabolic, hematologic, immunologic, other congenital or genetic, malignant, or renal abnormalities.
Labs	
SARS-CoV-2 real-time RT-PCR platforms	Xpert® Xpress CoV-2 plus test (Cepheid, Sunnyvale, CA, USA), multiplex panel Xpert® Xpress CoV-2/Flu/RSV plus test, Panther Fusion® SARS-CoV-2 Assay (Hologic, Marlborough, MA, USA), and Cobas® SARS-CoV-2 Qualitative (Roche, Basel, Switzerland).
Abnormal UA and urine microscopy	Positive leukocyte esterase and/or ≥5 WBCs/hpf
Infections	
Urinary tract infection	The urine collection method was captured. Bacterial growth on urine culture when collected by perineal bag was confirmed by catheterized specimen to be defined as abnormal. Infants 7-60 days: isolation of a single urinary bacterial pathogen with colony count ≥ 10,000 CFU/mL and associated leukocyte esterase or pyuria (≥5 WBCs/hpf) detected on UA. Infants 61-90 days: isolation of a single urinary bacterial pathogen with colony count ≥50,000 CFU/mL and associated leukocyte esterase or pyuria detected on UA.
Bacteremia	Bacteremia was defined as the isolation of a bacterial pathogen from blood. Coagulase-negative *Staphylococcus*, viridans group *Streptococcus*, *Micrococcus* species, and diphtheroids were considered contaminants.
Bacterial meningitis	“Definite” with the isolation of a single bacterial pathogen from CSF, or as “probable” with sterile CSF with pleocytosis obtained after antibiotics, with diagnosis confirmed by a pediatric infectious disease specialist (TLG). Infants who did not undergo lumbar puncture and did not receive therapy for bacterial meningitis were presumed not to have meningitis.

Our primary outcome was an ED diagnosis of UTI, bacteremia, or bacterial meningitis (Table 1). Data on age, sex, race/ethnicity, gestational age at delivery, presence of underlying comorbidities, maximum temperature in a medical setting, laboratory studies, and antibiotic prescriptions were extracted from EHRs. Race and ethnicity were included to demonstrate that the diversity of the cohort reflects the diversity of the population of northern California, and were self-reported as Asian or Pacific Islander, Black, Hispanic or Latino, or non-Hispanic White individuals. Regular expression-based text parsing was performed in the Statistical Analysis System (SAS) to generate text snippets informing physician adjudication (TLG) of the variables: maximum home temperature and ill appearance. Manual chart review was used to confirm COVID-19 diagnoses and home test results. We queried the EHR for return visits within seven days associated with missed UTI or IBI.

Statistical analysis

Bivariate associations were tested using the Wilcoxon rank-sum test, or the exact test for counts less than 5. All statistical tests were two-sided, and p<0.05 was considered statistically significant. Both SAS 9.4 (SAS Institute Inc., Cary, NC, USA) and R Studio version 4.2.1 (RStudio: Integrated Development Environment for R, Boston, MA, USA) were used as statistical software.

## Results

**Figure 1:**
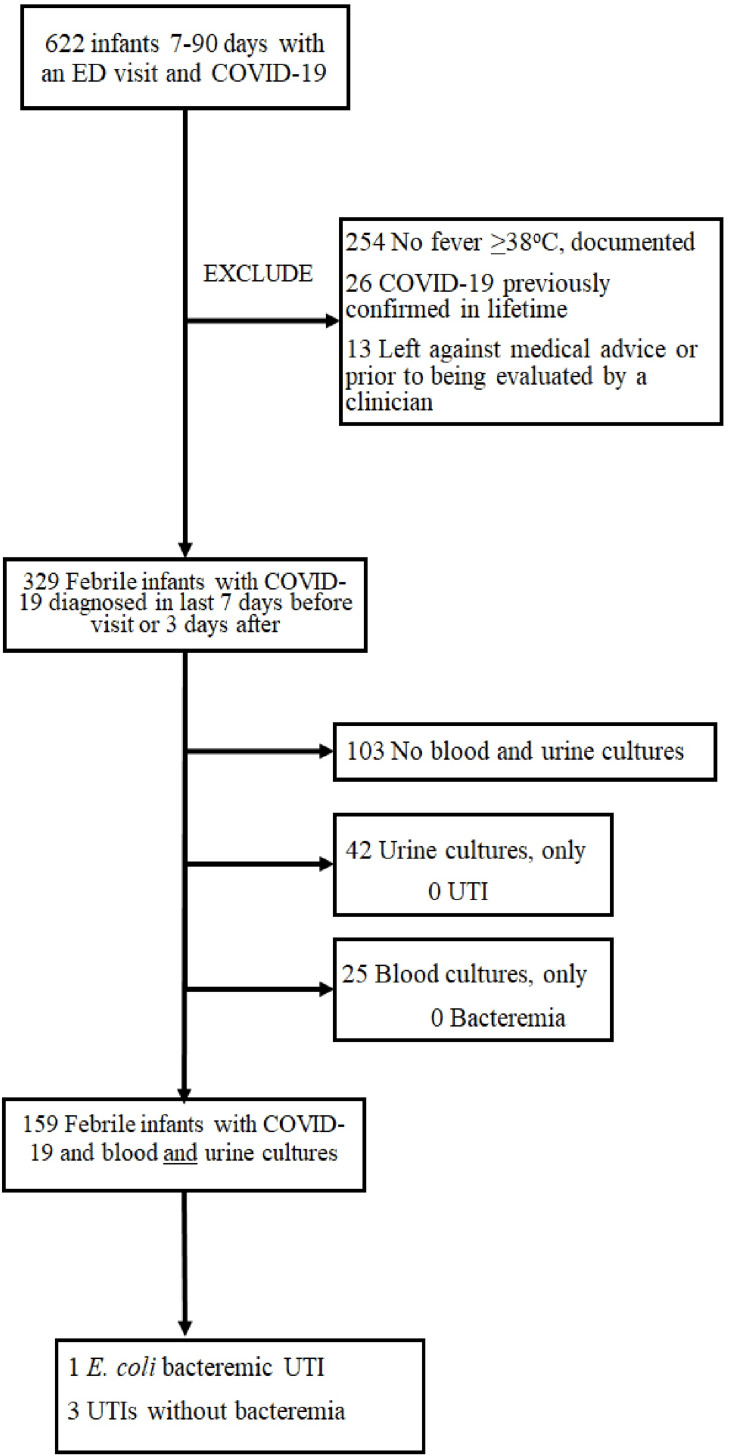
Flow diagram illustrating selection of infants with COVID-19 ED: emergency department; UTI: urinary tract infection; *E. coli*: *Escherichia coli*

Six hundred and twenty-two infants with COVID-19 were seen in the ED, with 606 (97%) confirmed by positive SARS-CoV-2 RT-PCR, and the remainder by parental report of positive home antigen testing. Two hundred and ninety-three infants were excluded for lack of documented fever, diagnosis of COVID-19 more than seven days prior to the ED visit, or leaving the ED against medical advice or prior to being evaluated by a clinician (Figure 1). Table 2 shows the demographics of the 329 febrile infants with COVID-19.

**Table 2:** Characteristics of febrile infants with COVID-19 IQR: interquartile range; ED; emergency department

Characteristics (N = 329)	Values
Age, median (IQR), (days)	46.2 (22.6)
Age category (days), n (%)	
7-21	48 (15)
22-28	26 (7.9)
29-60	174 (53)
61-90	81 (25)
Female, n (%)	155 (47)
Race/Ethnicity, n (%)	
Asian/Native Hawaiian/Other Pacific Islander individuals	53 (16)
Black or African American individuals	25 (8)
Hispanic or Latino individuals	115 (35)
Missing or Decline to state	37 (11)
White individuals	99 (30)
Prematurity, n (%)	21 (6.4)
Underlying comorbidity, n (%)	17 (5.2)
Maximum ED temperature (°C), mean (SD)	38.1 (0.6)
Repeat ED visit within 7 days, n (%)	31 (9.4)

Evaluations of infants differed by age and year of COVID-19 infection, with lower rates of complete evaluation (e.g., urine, blood, and CSF) in older infants and those presenting later in the pandemic. Two hundred and one infants had urine cultures, 184 had blood cultures, 159 had both urine and blood collected, and 12 had urine, blood, and CSF collected (Figure 1 and Table 3). Of the 201 infants with urine collected, four (2% (95% confidence interval (CI) 0.54%, 5.02%)) had a UTI (Table 4). Contaminated blood cultures were more common than bacteremia. Of the 184 infants with blood cultures obtained, one grew a pathogen, and 16 (8.7%) had contaminated blood cultures. Of the 16 infants with contaminated blood cultures, six were asked to return to the ED, including four admitted to the hospital due to blood culture positivity. Contaminated blood cultures prompted 11 additional blood cultures and three additional lumbar punctures. One of the 159 infants (age 49 days) (0.6% (95% CI 0.00%, 3.0%)) with both blood and urine collected had an *Escherichia coli* bacteremic UTI. There were no cases of isolated bacteremia. Only 12 infants had CSF obtained; however, no infants received treatment for bacterial meningitis (0/329 (95% CI 0.00%, 1.12%)).

**Table 3:** Urine, blood, and CSF cultures obtained by age in febrile infants with COVID-19 CSF: cerebrospinal fluid

Age (days)	Urine + Blood + CSF	Urine + Blood	Urine only	Blood only	None
7-21	9	26	0	7	6
22-28	3	18	0	2	3
29-60	0	90	24	14	46
61-90	0	13	18	2	48
Total	12	147	42	25	103

**Table 4:** Descriptors of four febrile infants with UTI and COVID-19 UTI: urinary tract infection; ANC: absolute neutrophil count; CFU/mL: colony-forming units per milliliter; hpf: high-powered field; UA: urinalysis; WBC: white blood cell; *E. coli*:* Escherichia coli*

Characteristics	Infant 1	Infant 2	Infant 3	Infant 4
Age (days)	12	26	49	67
Sex	Female	Male	Male	Male
Race/Ethnicity	Latino	Asian	Latino	Latino
Maximum temperature (°C)	38.3	38.2	39.2	39.6
Laboratory studies				
Blood WBC (K/µL)	9.6	4.4	16.9	19.7
Blood ANC (K/µL)	2.3	1.3	9.3	9.1
UA leukocyte esterase (/µL)	Small	Large	Large	Moderate
UA nitrite (mg/dL)	No	Yes	No	Yes
UA WBC (/hpf)	7	>182	>182	27
Urine pathogen (CFU/mL)	10-50,000 *E. coli*	10-50,000 *E. coli*	50-100,000 *E. coli*	>100,000 *E. coli*
Concomitant bacteremia	No	No	Yes	No
Duration of hospitalization (days)	4	4	2	0

Infants with both blood and urine collected were younger than the full febrile infant cohort (mean age 37 days vs. 46 days) (Table 5). Factors associated with UTI were higher blood white blood cell (WBC) (p=0.001) and absolute neutrophil counts (ANC) (p=0.036), and abnormal urinalysis (UA) and urine microscopy (p<0.001). There were no differences in age, sex, race/ethnicity, past medical history, maximum temperature, or appearance. A single infant with both blood and urine collected was ill appearing at presentation and did not have a UTI or IBI.

**Table 5:** UTI in 159 febrile infants with COVID-19 with both blood and urine cultures IQR: interquartile range; UTI: urinary tract infection; ANC: absolute neutrophil count; hpf: high-powered field; UA: urinalysis; WBC: white blood cell

Characteristics	Overall, N = 159	Without UTI, N = 155	With UTI, N = 4
Age, median (IQR), (days)	37.2 (19.5)	37.2 (19.5)	39.3 (24.2)
Age category (days), n (%)			
7-21	35 (22)	34 (22)	1 (25)
22-28	21 (13)	20 (13)	1 (25)
29-60	90 (57)	89 (57)	1 (25)
61-90	13 (8.2)	12 (7.7)	1 (25)
Sex, n (%)			
Female	73 (46)	72 (46)	1 (25)
Male	86 (54)	83 (54)	3 (75)
Race/Ethnicity, n (%)			
Asian/Native Hawaiian/Other Pacific Islander	24 (13)	23 (13)	1 (25)
Black/African American individuals	12 (7.5)	12 (7.7)	0 (0)
Hispanic/Latino individuals	58 (36)	55 (35)	3 (75)
Missing/Decline to state	15 (9.4)	15 (9.7)	0 (0)
White individuals	50 (31)	50 (32)	0 (0)
Prematurity, n (%)	10 (6.3)	10 (6.5)	0 (0)
Congenital anomaly, n (%)	9 (5.7)	9 (5.8)	0 (0)
Maximum temperature > 38.5°C, n (%)	46 (29)	44 (28)	2 (50)
Ill appearance, n (%)	1 (0.6)	1 (0.6)	0 (0)
Laboratory studies			
Blood WBC (K/µL), n (%)			
<5	43 (27)	42 (27)	1 (25)
5-15	106 (67)	105 (68)	1 (25)
>15	3 (1.9)	1 (0.6)	2 (50)
Missing	7 (4.4)	7 (4.5)	0 (0)
Blood ANC (K/µL), n (%)			
<5.2	140 (88)	138 (89)	2 (50)
>5.2	10 (6.3)	8 (5.2)	2 (50)
Missing	9 (5.7)	9 (5.8)	0 (0)
UA leukocyte esterase, n (%)			
Abnormal	12 (7.5)	9 (5.8)	4 (100)
Normal	137 (86)	137 (88)	0 (0)
Missing	10 (6.3)	9 (5.8)	0 (0)
UA WBC (/hpf), n (%)			
<5	28 (18)	28 (18)	0 (0)
>5	11 (6.9)	8 (5.2)	4 (100)
Missing	120 (75)	119 (77)	0 (0)

Return visits to the ED within seven days of an index visit were common and occurred in 31 of 329 infants (9%). Reasons for return visits included clinical concerns and bacterial growth (pathogenic and non-pathogenic) of blood and urine cultures. None of the 103 infants without urine or blood cultures received antibiotics or returned to the ED with a missed UTI or IBI. This included a single ill appearing infant who was not found to have a subsequent bacterial infection.

## Discussion

In this retrospective study of young, febrile infants with COVID-19, rates of UTI and IBI were low. Febrile infants with COVID-19 were at very low risk for IBI but did have some risk for concomitant UTI. Of those tested in our cohort, 2% (95% CI 0.54%, 5.02%) had a UTI, 0.5% (95% CI 0.00%, 3.0%) had bacteremia, and 0 (95% CI 0.00%, 1.12%) had bacterial meningitis.

The rates of UTI and bacteremia are likely an overrepresentation of actual incidence, as there was no evidence that infants who did not receive an ED evaluation had either. Including all febrile infants, the UTI rate decreased to 1.2%. Importantly, there were no cases of isolated bacteremia or bacterial meningitis. The risk of a missed IBI is quite low, as no infant received treatment for bacterial meningitis, and all infants were followed for seven days after the index ED visit.

Only 3.6% of febrile infants underwent a full evaluation, compared to nearly one-third (31.3%) who had no studies performed. This suggests that ED clinicians were reassured that COVID-19 alone was the source of the fever. As we gathered data [3-5,8-10,15-17] and experience during the pandemic, the rates of IBI were low in febrile infants with COVID-19, and ED clinicians began obtaining even fewer studies.

We used the narrow AAP CPG definition of UTI, which requires an abnormal UA [18]. Our rates of UTI closely mirror both Aronson et al. [7] and Burstein et al. [17], who published larger studies on infants aged 8-60 days who fit the inclusion and exclusion criteria for the AAP CPG [18] and used the narrower AAP CPG definition of UTI. If a UTI was also defined as an abnormal urine culture with a single pathogen of >50,000 CFU/mL with a normal UA, our rates of UTI were 4%, which is comparable to other publications of smaller cohorts of young, febrile infants with COVID-19 [8-10]. Regardless of the definition used, UTI rates are low compared with infants without COVID-19 [7-10,17].

The single factor associated with concomitant bacterial infection in our cohort of febrile infants with COVID-19 was abnormal UA with microscopy. Every infant with a concomitant bacterial infection had a UTI, including the only patient with an IBI who had an *E. coli* bacteremic UTI. As seen in infants without COVID-19 [18], higher blood WBC counts and ANCs were more common in infants with UTI than those without UTI. Low blood WBC counts (e.g., <5 K/µL) were not uncommon in those with COVID-19.

Only a single infant in our cohort was found to have bacteremia, and there were no cases of bacterial meningitis. Frequency of bacteremia and bacterial meningitis in febrile infants with COVID-19 was very low in other smaller and larger published cohorts of infants [4,5,7-17]. Unfortunately, blood culture acquisition was not without harm, as blood culture contamination is not an uncommon occurrence [19]. Contaminated blood cultures outnumbered bacteremia 16:1 and led to unnecessary ED visits, hospital admissions, repeat blood cultures, and lumbar punctures.

Guidelines are needed to address the evaluation of febrile infants with COVID-19. Clinicians vary in their level of acceptable risk when determining an appropriate evaluation. As supported by our study and other published reports [7-17], febrile, well-appearing infants aged 7-60 days have a non-negligible risk of UTI. Considering their increased risk of UTI complications, we recommend, at a minimum, that they undergo a UA with microscopy to evaluate for concomitant bacterial infections. In infants older than two months, as the risk of UTI is between 2% and 5%, obtaining a UA with microscopy should be at a clinician’s discretion [20,21].

Rates of concomitant UTI and IBI appear comparable with COVID-19 infection and bronchiolitis. Studies included in a bronchiolitis meta-analysis found a range of UTI rates, with the overall estimate being 3.1%, which decreased to 0.8% when UA was included as part of the UTI diagnosis [22]. Cases of IBI were comparably exceedingly low.

Ill appearance was rare in our cohort of febrile infants with COVID-19, as only 2 of 329 (0.6%) were ill appearing at presentation. Neither was found to have a UTI or IBI. As the percentage of infants with ill appearance was surprisingly low, no conclusions can be drawn as to the appropriate management or rates of bacterial infection in ill-appearing infants. The low rates of ill appearance might be attributed to documentation bias if clinicians were aware of an infant's COVID-19 status. However, given that physical exams are generally conducted before lab results, this bias is unlikely to have substantially influenced the documented findings.

Our study has some limitations. We studied infants with a positive SARS-CoV-2 RT-PCR or COVID-19 diagnosis. Obtaining SARS-CoV-2 RT-PCR testing on all febrile young infants was standard of care during the pandemic; however, it is possible that a small percentage of infants did not have SARS-CoV-2 testing. We did not examine bacterial infections in young infants testing negative for SARS-CoV-2 during this study period, and so are unable to make direct comparisons of rates of UTI and IBI between infants with and without COVID-19. The outcomes of UTI and IBI were low, limiting the ability to develop predictors of concomitant bacterial infection. However, this study adds to the body of literature that febrile infants with COVID-19 are low risk for UTI and IBI.

## Conclusions

Testing for COVID-19 should remain available to febrile infants presenting to the ED, as concomitant UTI and IBI are uncommon in these infants, and management should differ compared to those without viral infection. Given the rarity of IBI in febrile infants with COVID-19, most infants do not need to be hospitalized or undergo numerous diagnostic tests, as an abnormal UA and blood WBC or ANC predict UTI and IBI. Based on our study and other published reports, we recommend, at a minimum, that all febrile well-appearing infants aged 7-60 days with COVID-19 be evaluated for concomitant bacterial infections with a UA with microscopy.
